# Short‐term local control after VATS segmentectomy and lobectomy for solid NSCLC of less than 2 cm

**DOI:** 10.1111/1759-7714.13766

**Published:** 2020-12-03

**Authors:** Marc Darras, Amaya Ojanguren, Céline Forster, Matthieu Zellweger, Jean Yannis Perentes, Thorsten Krueger, Michel Gonzalez

**Affiliations:** ^1^ Service of Thoracic Surgery Lausanne University Hospital (CHUV) Lausanne Switzerland; ^2^ Faculty of Biology and Medicine University of Lausanne (UNIL) Lausanne Switzerland

**Keywords:** Lobectomy, lung cancer, lymph node dissection, segmentectomy, VATS

## Abstract

**Introduction:**

VATS pulmonary segmentectomy is increasingly proposed as a parenchyma‐sparing resection for tumors smaller than 2 cm in diameter. The aim of this study was to compare short‐term oncological results and local control in solid non‐small cell lung cancers (NSCLCs) <2 cm surgically treated by intentional VATS segmentectomy or lobectomy.

**Methods:**

This study was a single center retrospective study of consecutive patients undergoing VATS lobectomy (VL) or segmentectomy (VS) for solid <2 cm NSCLC from January 2014 to October 2019.

Results

In total, 188 patients with a median age of 65 years (male/female: 99/89) underwent VS (*n* = 96) or VL (*n* = 92). Segmentectomies in the upper lobes were performed in 57% and as a single segment in 55% of cases. There was no statistically significant difference between VS and VL in terms of demographics, comorbidities, postoperative outcomes, dissected lymph node stations (2.89 ± 0.95 vs. 2.93 ± 1, *P* = 0.58), rate of pN1 (2.2% vs. 2.1%, *P* = 0.96) or pN2 upstaging (1.09% vs. 1.06%, *P* = 0.98). Adjuvant chemotherapy was given in 15% of patients in the VL and 11% in the VS group. During follow‐up (median: 23 months), no patients presented with local nodal recurrence or on the stapler line (VS group). Three patients on VL and two in VS groups presented with recurrence on the remnant operated lung. New primary pulmonary tumors were diagnosed in 3.3% and 6.3% of patients in the VL and VS groups, respectively.

**Conclusions:**

Despite the short follow‐up, our preliminary data shows that local control is comparable for VATS lobectomy and VATS segmentectomy for patients with NSCLC <2 cm.

## Graphical abstract

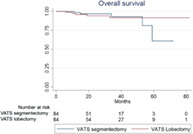

This study analyzes the short‐term oncological results and local control of solid NSCLC smaller than 2 cm, surgically treated by intentional VATS segmentectomy or lobectomy. Despite the short follow‐up, our preliminary data shows that local control is comparable for VATS lobectomy and VATS segmentectomy for patients with NSCLC <2 cm.

## Introduction

Lobectomy plus mediastinal lymph node dissection has become the standard surgical procedure for the treatment of patients with operable NSCLC.^1^, ^2^ Historically, sublobar resections such as segmentectomy or wedge resections have been used as an alternative surgical option for patients with compromised lung function ineligible for lobectomy,^3^, ^4^ or in elderly patients, in whom segmentectomy is associated with lower complication rates.^5^ However, several factors have sparked a debate on the extent of lung resection with curative intent. First, the advancement in CT screening programs for early lung cancer detection increased the number of clinically suspicious nodules compatible with early‐stage lung cancer and second, the minimally invasive techniques for lobectomy and segmentectomy have been consolidated during the last decade. Nevertheless, VATS pulmonary segmentectomy is a technically challenging procedure: it requires individual dissection of segmental bronchovascular structures and identification of intersegmental planes to prevent incomplete resections and postoperative complications. Both aspects have ignited the controversy on whether segmentectomies are indicated for patients with early‐stage lung cancer as intentional resection and not only for those with limited lung function. Since patients with smaller tumors have a better prognosis, sublobar resections should suffice.^6^

One randomized controlled trial addressed this question and reported a preference for lobectomy in terms of local control and survival.^7^ However, recent studies indicate that segmentectomy could achieve recurrence and survival rates comparable to lobectomy for early stage T1N0M0 NSCLC ≤2 cm, as long as adequate surgical margins and systematic lymph node dissection are performed.^8^, ^9^ In addition, many studies have included pure ground‐glass opacity (GGO) nodules to be candidates for segmentectomy. This would lead to a selection bias as GGO lesions are less aggressive, and thus present better oncological prognosis.^10^, ^11^

The aim of this retrospective monocentric study was to compare short‐term oncological results and local control in solid early stage NSCLC ≤2 cm treated by intentional VATS segmentectomy or lobectomy.

## Methods

### Patients

We retrospectively reviewed the records of all patients who underwent VATS pulmonary lobectomy or segmentectomy for solid early clinical stage cT1a‐b cN0 NSCLC from January 2014 to October 2019 in the Lausanne University Hospital. The Local Ethics Committee approved the study and individual consent was waived (Referral number: 2020–02159). This study was reported according to the STROBE criteria for observational studies.^12^

Since January 2014, the resection extent for solid nodules <20 mm in diameter and proven or suspected to be NSCLC was decided based on various criteria. The study specifically included solid or mixed lesions with a solid component of more than 5 mm. All cases were individually discussed by a multidisciplinary board. Patients were assessed by chest computed tomography (CT) and fluoro‐deoxy‐glucose (FDG) positron emission tomography (PET) scans. Clinical stage was assessed with preoperative imaging and staging procedures. Tumor size and maximum standard uptake values (SUVmax) were determined. All patients with suspected lymph node involvement on preoperative imaging underwent endobronchial ultrasound (EBUS) fine‐needle aspiration or mediastinoscopy before surgery. All eligible patients underwent a transthoracic or bronchoscopic biopsy to determine the histology of the pulmonary lesion when technically possible. A VATS segmentectomy was suggested to patients with a suspected solid NSCLC <2 cm peripherally located in a specific segment, if the minimal surgical margin was 2 cm.

Definitive pathological T‐stage was defined by measuring the maximum diameter of the tumor's invasive component. Patients with histology of adenocarcinoma, squamous cell, large cell carcinoma and carcinoid tumor were included in this study. Benign lung disease was defined as an exclusion‐criteria. Lesions that were radiologically identified as GGO were also excluded. All data for the pathological information was extracted from published reports. Pathological variables included tumor size and histology, TNM stage (seventh edition), number and localization of dissected lymph nodes as well as pathological upstaging. Adjuvant chemotherapy was indicated for patients with histologically confirmed N1–N2 disease. In addition to patients with nodal involvement, chemotherapy was also discussed for patients with T3 and T2 tumors with visceral pleural invasion. In some cases, chemotherapy was not administered due to patient refusal or comorbidities. Patients undergoing wedge resection, bilobectomy, concurrent lobectomy and segmentectomy or pneumonectomy were excluded. Patients requiring a conversion thoracotomy or completion lobectomy for incomplete resection during segmentectomy or patients who received neoadjuvant treatment were also excluded.

### Surgical technique

Anatomical segmental resection was accomplished by removing one or more pulmonary segments to achieve complete resection. VATS segmentectomy was performed under general anesthesia with lung isolation by double lumen intubation. Surgical resection was undertaken using a standardized three‐port or one port approach since 2018 (utility incision in the fourth intercostal space, one for 10 mm 30° thoracoscope in the seventh intercostal space anteriorly and one posteriorly). Segmentectomy was performed with individual dissection of the segmental bronchus, arteries and veins. All bronchovascular structures were transected using endoscopic staplers or energy devices. The intersegmental plane was defined using indocyanine green when necessary and divided using staplers. In all cases systemic hilar and mediastinal nodal dissection was performed. Frozen section was performed for suspected hilar lymph nodes and completion lobectomy undertaken in cases of lymph node involvement. All surgical specimens were extracted in a protective bag to prevent chest wall seeding of malignant disease.

After discharge chest CT scans were performed every three months for the first two years then every six months for a total of five years. Locoregional recurrence was defined as any recurrence in the ipsilateral lung, hilum, or mediastinum without evidence of distance metastasis for patients who underwent anatomic segmentectomy and lobectomy. Distant recurrence was defined as the presence of a contralateral mediastinum or lung, or any extra‐thoracic metastatic disease.

All data was prospectively collected from our database. Individual medical records were retrospectively reviewed and analyzed for patient demographics, pulmonary function, type of lobectomy and segmentectomy, histological findings, lesion size and localization, operative time, surgical outcome, postoperative morbidity and mortality. The primary endpoint was overall survival (OS), defined as the time from surgery to either death or last follow‐up.

### Statistical analysis

The baseline characteristics and clinical outcomes were described using numbers and percentages for categorical variables and median and range for continuous measurements. Comparison of peri‐ and postoperative variables between lobectomy and segmentectomy were analyzed with Fisher's exact test for categorical variables, and Mann‐Whitney U test for continuous variables. The event was considered to be any death in order to obtain overall survival (OS) at three years. Patients were censored at the time of last follow‐up. Log‐rank tests were used to compare differences in Kaplan‐Meier estimates. A two‐tailed hypothesis was used and significance accepted if *p* < 0.05. All analyses were performed using STATA software, version 14 (StataCorp LLC, Texas, USA).

## Results

### Demographics

Between January 2014 and October 2019, 188 patients (male/female: 99/89; mean age 64.95 ± 10.5 years) underwent anatomical pulmonary VATS segmentectomy (VS, *n* = 96) or VATS lobectomy (VL, *n* = 92) for cT1a‐b cNo NSCLC. Patient characteristics are summarized in Table 1. Patients were slightly older in the VS group, although not significantly (*P* = 0.09). There was no statistical difference between groups in preoperative pulmonary functions, associated comorbidities or ASA score.

**Table 1:** Patient characteristics

	Total (*n* = 188)	VATS lobectomy (*n* = 92)	VATS segmentectomy (*n* = 96)	*P*‐value
Sex (male/female)	99/89	50/42	49/47	0.65
Age (mean)	64.95 ± 10.5	63.6 ± 10.6	66.2 ± 10.3	0.09
BMI (mean)	25.2 ± 4.7	25.1 ± 5.1	25.4 ± 4.3	0.71
FEV1 (mean)	86.6 ± 21	87.5 ± 19	85.7 ± 22	0.56
DLCO (mean)	75.8 ± 20	77.9 ± 19	73.8 ± 21	0.18
Cardiopathy	20 (11%)	7 (8%)	13 (14%)	0.18
HTA	84 (45%)	35 (38%)	49 (51%)	0.07
Atrial fibrillation	23 (12%)	13 (14%)	10 (10%)	0.44
COPD	84 (45%)	37 (40%)	47 (49%)	0.23
Tobacco	185 (98%)	77 (84%)	78 (81%)	0.66
Diabetes	29 (15%)	13 (14%)	16 (17%)	0.63
Kidney failure	15 (8%)	5 (5%)	10 (10%)	0.35
Immunosuppression	4 (2%)	2 (2%)	2 (2%)	0.97
Previous cancer	81 (43%)	32 (35%)	49 (51%)	0.02
ASA score				
I	3 (1%)	1 (1%)	2 (2%)	0.76
II	99 (53%)	51 (55%)	48 (50%)	
III	85 (45%)	39 (42%)	46 (48%)	
IV	1 (0.5%)	1 (0.5%)	0 (0%)	

### Surgery

In the VL group, upper lobectomies were performed in 58% and in the right side in 75% of cases. In the VS group, segmentectomies were accomplished in the upper lobes in 57% and as a single segment in 55% of cases. The majority of segmentectomies were carried out in the left side (60%). Table 2 summarizes the surgical aspects. We observed a statistical difference in the absolute mean number of lymph nodes towards the VL group (14.9 ± 7.9 vs. 9.4 ± 7.1; *P* < 0.001). There was no statistically significant difference between the overall number of dissected lymph node stations in the VL and VS groups (2.89 ± 0.95 vs. 2.93 ± 1 [*P* = 0.58]), nor in the specific area (hilar or mediastinal lymph nodes).

**Table 2:** Pathological and surgical results

	Total (n = 188)	Lobectomy (*n* = 92)	Segmentectomy (*n* = 96)	*P*‐value
Lobectomy	92			
Right upper lobe		40 (43%)		
Right middle lobe		19 (21%)		
Right lower lobe		10 (11%)		
Left upper lobe		14 (15%)		
Left lower lobe		9 (10%)		
Segmentectomy	96			
Upper lobe			55 (57%)	
Lower lobe			41 (43%)	
Single			53 (55%)	
Multiple			43 (45%)	
Seg 1			12 (13%)	
Seg 1,2			6 (6%)	
Seg 1,3			2 (2%)	
Seg 1–3			15 (16%)	
Seg 2			6 (6%)	
Seg 3			3 (3%)	
Seg 4–5			11 (11%)	
Seg 6			28 (29%)	
Seg 8			4 (4%)	
Seg 9–10			2 (2%)	
Seg 7–10			7 (7%)	
Adenocarcinoma	134 (71%)	62 (67%)	72 (75%)	0.25
Squamous cell carcinoma	33 (18%)	17 (18%)	16 (17%)	
Large cells tumor	6 (3%)	4 (4%)	2 (2%)	
Carcinoid tumor	15 (8%)	9 (10%)	6 (6%)	
Lymph nodes dissected (number)	12.1 ± 7.93	14.9 ± 7.9	9.4 ± 7.1	<0.0001
Areas dissected (number)	2.89 ± 0.95	2.84 ± 0.9	2.93 ± 1	0.51
Hilar area (number)	0.75 ± 0.43	0.76 ± 0.42	0.73 ± 0.44	0.58
Mediastinal area (number)	2.16 ± 0.8	2.1 ± 0.7	2.2 ± 0.9	0.39
Pathological stage				
T1a (<1 cm)	40 (21%)	16 (17%)	24 (25%)	0.29
T1b (1–2 cm)	120 (64%)	57 (62%)	63 (66%)	
T2 (visceral pleural invasion)	23 (12%)	17 (18%)	6 (6%)	
T3 (satellite nodule, parietal pleural invasion)	5 (3%)	2 (2%)	3 (3%)	
N0	182 (97%)	89 (97%)	93 (97%)	0.95
N1	4 (2%)	2 (2%)	2 (2%)	
N2	2 (1%)	1 (1%)	1 (1%)	
PET‐CT uptake	4.56 ± 3.69	5.46 ± 4.11	3.7 ± 3.02	0.021
Adjuvant chemotherapy	25 (13.2%)	14 (15%)	11 (11%)	0.44

### Pathological analysis

Definitive pathological analysis showed a majority of adenocarcinoma (134 patients, 71%) with no difference between VL and VS (67% vs. 75%; *P* = 0.25). The remaining tumors were squamous cell carcinoma, large cells tumors and carcinoid tumors and were equally distributed between both groups. Tumor upstaging was found in 19 VL (20%) and nine VS patients (9%), due to either invasion of visceral/parietal pleura, or the finding of an additional nodule in the specimen. Nodal upstaging to N1 or N2 was observed in three patients (2%) in each group, with a specific rate of pN1 (2.2% vs. 2.1%, *P* = 0.96) and pN2 upstaging (1.09% vs. 1.06%, *P* = 0.98).

### Perioperative results

The mean operation time was 141 ± 31 minutes, with no statistical difference between lobectomies and segmentectomies (144 minutes vs. 138 minutes; *P* = 0.28). The overall 30‐day mortality and morbidity were 0% and 28%. Postoperative outcomes were similar between both groups. Postoperative complications were mainly minor cardiopulmonary complications, without intergroup differences. Seven patients required reoperation for empyema (*n* = 2), hemothorax (*n* = 1), pneumothorax (*n* = 2) and subcutaneous emphysema (*n* = 2). The mean duration of thoracic drainage was three days in the lobectomy group and two days in the segmentectomy group (*P* = 0.28). Mean postoperative length of stay was six days in the lobectomy group and seven days in the segmentectomy group (*P* = 0.33). Table 3 summarizes perioperative outcomes.

**Table 3:** Perioperative outcomes

	Overall (*n* = 188)	Lobectomy (*n* = 92)	Segmentectomy (*n* = 96)	*P*‐value
Operative mean time	2 hours 21 minutes	2 hours 24 minutes	2 hours 18 minutes	0.28
Mortality	0	0	0	1
Overall morbidity	62 (32%)	24 (26%)	38 (40%)	0.05
Cardiopulmonary complications	52 (28%)	21 (23%)	31 (32%)	0.13
Reoperation	7 (4%)	2 (2%)	5 (5%)	0.263
Readmission	11 (6%)	7 (8%)	4 (4%)	0.32
Duration of drainage (median) (days) (IQR)	2 (1–4)	3 (1–4.5)	2 (1–4.5)	0.28
Postoperative duration of stay (median) (days) (IQR)	6 (4–9)	6 (4–9)	7 (4–9.5)	0.33

### Recurrence and survival

**Figure 1:**
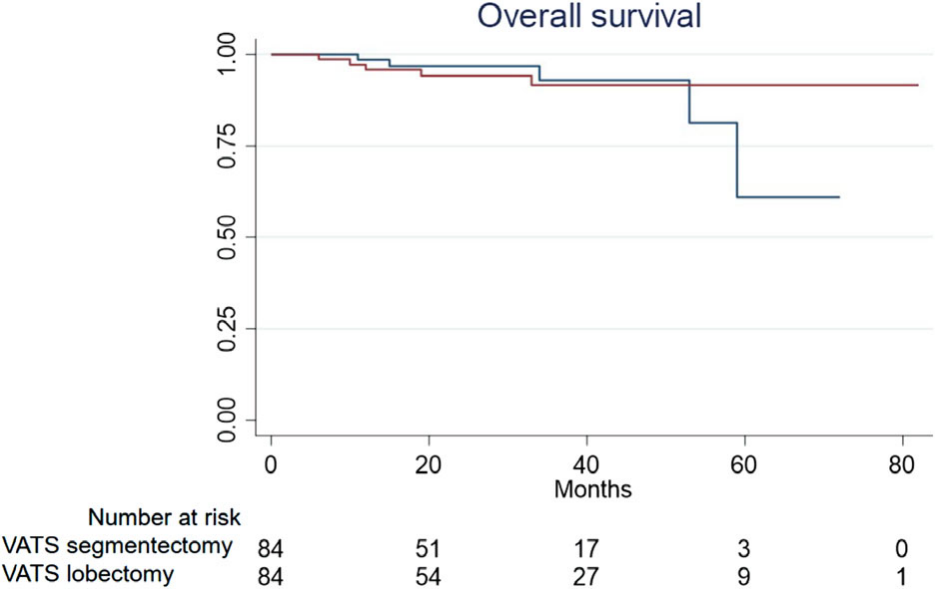
Overall survival analysis between VATS lobectomy and segmentectomy (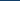 ) VATS segmentectomy (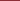 )VATS lobectomy.

During follow‐up (median 23 months, IQR: 6–38.5), 20 patients (11%) were lost hence censored. The follow‐up was statistically longer in theVL group since our VATS segmentectomy program was launched in 2016. No VS patients presented local nodal recurrence or on the stapling line. Five patients (three VL and two VS; *P* = 0.388) presented recurrence on the remnant operated lung. Distant recurrences were diagnosed in 6.5% of VL cases and 2% of VS cases (*P* = 0.124). During follow‐up, nine patients (six VL and three VS) developed metachronous tumors in other lobes or lung, presenting different histological subtypes. Other primary tumors appeared in 14 patients (7.4%), requiring further treatment. During follow‐up, death occurred in five VL patients (5.6%) (three lung cancer recurrences and two other primary cancers) and in five VS patients (5.2%) (four other primary cancers and one cardiac problem) (*P* = 0.945). Table 4 presents the follow‐up data. The estimated survival at three years was 93% in the VS group and 92% in the VL group (log rank 0.738) (Fig 1).

**Table 4:** Long‐term follow‐up

	Overall (*n* = 188)	Lobectomy (*n* = 92)	Segmentectomy (*n* = 96)	*P*‐value
Lost to follow‐up	20 (10.7%)	8 (8.7%)	12 (12.5%)	0.049
Follow‐up (months) (IQR)	23 (6–38.5)	26 (11–51)	21 (3–34)	
Local recurrence				
Lymph node	0 (0%)	0 (0%)	0 (0%)	1
Lung	5 (2.7%)	3 (3.3%)	2 (2.1%)	0.388
Distant recurrence	8 (4.3%)	6 (6.5%)	2 (2%)	0.124
New lung cancer	9 (4.8%)	3 (3.3%)	6 (6.3%)	0.345
Other primary cancer	14 (7.4%)	5 (5.4%)	9 (9.4%)	0.300
Death	10 (5.3%)	5 (5.4%)	5 (5.2%)	0.945

## Discussion

The optimal resection extent for early stage non‐small cell lung cancer (NSCLC) <2 cm is currently under debate. Lobectomy is considered the gold standard, yet anatomical pulmonary segmentectomy has gained acceptance during the last decade for patients with compromised cardiopulmonary function. Lung cancer screening programs have led to the improved detection of small nodules or ground‐glass opacities. Consequently, the debate about the efficacy of lobectomy versus sublobar resection for early stage NSCLC has been revisited. The main advantage of segmentectomy over lobectomy is that it spares more parenchyma, and thus better preserves lung function.^13^, ^14^

The debate pertains to whether or not anatomic segmentectomy is comparable to lobectomy in terms of oncological outcomes in patients with stage I NSCLC. In 1995, a randomized trial compared sublobar resection with lobectomy for clinical early stage NSCLC.^7^ This trial concluded that sublobar resection was associated with high rates of recurrence and death. However, the study included 40 wedge resections (out of 122) in the sublobar group, which were not analyzed separately from segmentectomy. In addition, this study did not specifically study tumors that were <2 cm.

Our study revealed no significant differences between VS and VL patients for early stage NSCLC smaller than 2 cm in terms of the number of dissected lymph node stations, rate of pN1 or pN2 upstaging, local recurrence or survival. Therefore, as far as the oncological results are concerned, segmentectomy and lobectomy are comparable and neither procedure can be viewed as superior to the other.

There is conflicting data in the literature regarding early‐stage NSCLC. A recent systematic review with meta‐analysis^15^ included 12 nonrandomized studies involving 8072 participants and compared oncological outcomes of lobectomy versus segmentectomy in NSCLC patients with clinical T1N0M0. It showed that segmentectomy was associated with shorter overall survival (OS), but identified comparable disease‐free survival (DFS) for these patients and patients with nodules ≤2 cm.^15^ However, when multivariate HRs were included, the impact of segmentectomy on OS and DFS was comparable to that of lobectomy in the entire cohort as well as in patients with nodules ≤2 cm. All studies included in this meta‐analysis were retrospective, which might impact its evidence level.

Similarly, another systematic review and meta‐analysis^16^ included 28 studies (26 retrospective, one prospective nonrandomized and a case‐matched study) and found that for stage I NSCLC, segmentectomy was inferior to lobectomy for OS, cancer‐specific survival (CSS) or recurrence‐free survival (RFS). The picture was different for stage IA <2 cm patients in whom no significant differences were found between segmentectomy and lobectomy for OS, CSS or RFS. The authors of these studies highlighted several possible sources of bias as follows: tumors were larger in the lobectomy group; intentional resections were performed for smaller, peripheral tumors; comorbidities were not comparable between groups; and compromised patients were more frequently selected for segmentectomy. For all the above‐mentioned reasons, the data reported by these authors should be carefully interpreted. Nonetheless, these results are corroborated by an older meta‐analysis^17^, ^18^ and by the SEER database^19^ where lobectomy was found to be superior to segmentectomy for stage IA lesions, but not for stage IA <2 cm after propensity score matching.

In contrast, our study did not show any difference between groups in terms of pulmonary function, comorbidities or ASA score. Moreover, since necessity segmentectomies were excluded, all VS patients underwent intentional resections, avoiding a major confounding factor. Moreover, all patient procedures were via a minimally invasive surgical approach. In this regard, it should be noted that most studies that compared segmentectomy versus lobectomy for early stage NSCLC failed to report, or to stratify their analysis by surgical approach.

Nowadays it is accepted that VATS is the preferable approach for early stage NSCLC.^20^, ^21^ It allows better visualization of the pleural cavity, generates less postoperative pain and morbidity, shortens hospital stays and causes a lesser inflammatory response compared to thoracotomy. This last element is particularly beneficial for oncology patients as a better compliance to adjuvant therapy is achieved.^22^

In terms of histopathological results, we found a nodal upstaging rate to N1 or N2 in 3,3% of patients. These results are similar to those reported for a VATS approach (3,6%),^23^ and lower than 7.9% reported for a full thoracoscopic approach,^9^ or 11.9% for an open approach.^24^ On the other hand, the debate is still ongoing as to whether minimally invasive systematic lymph node dissection is at least as efficient as open approach dissection.^25^, ^26^ Even if the number of sampled nodal stations does not differ between groups in our study, the number of lymph nodes dissected was significantly higher in the VL group. Such results could be explained by the surgical technique: during segmentectomies, we carried out a block dissection of the adenopathies together with the surrounding fatty tissue in an attempt to be more oncologically radical. As a result, the lymph nodes were not fragmented into smaller samples and the overall number was lower. In our opinion, intersegmental lymph node dissection is paramount for VS. It has been previously demonstrated by several authors^27^, ^28^ that local recurrence rates and overall survival are similar when lymph node clearance is performed during segmentectomy. In our study, no VS patients presented with local nodal recurrence or on the stapler line and only two patients in VS presented recurrence on the operated lung, at a distance from the resection site.

During follow‐up, death occurred in five VL patients (5.6%) (three lung cancer recurrences and two other primary cancers) and in five VS patients (5.2%) (four other primary cancers and one cardiac insufficiency). This is comparable to the results reported by a large meta‐analysis which compared segmentectomies and lobectomies, and reported no significant difference in overall survival in the intentionally selected group, although there was a worse outcome in the compromised group.^29^

Interestingly, we did not observe local recurrence on the stapler line in the VS group. Published data reports that pathologically negative margins, with a margin distance of 2 cm, are associated with minimal recurrence rates and survival identical to lobectomy for NSCLC <2 cm.^30^ We always follow the premise of maintaining a margin/tumor ratio > 1. This is critical when performing segmentectomies: an 85% of recurrence has been previously observed when the margin/tumor diameter ratios was <1.^31^ Therefore, to ensure complete segmental resection when the lesion crossed the intersegmental plane, an extended segmentectomy was performed with additional margins beyond the traditional segmental limits. When the lesion was located at the boundary between two segments, a combined segmentectomy or an extended wedge of the neighboring segment was chosen to achieve satisfactory margins. Finally, if there was any uncertainty about obtaining adequate margins, lobectomy was proposed. Thus, strictly pursuing the margin/tumor ratio > 1 goal has undoubtedly been key to avoid recurrences at the staple line in our series.

Some authors hypothesize that since segmentectomies require more extensive and deeper dissection into the hilum and division of the intersegmental plane, they may lead to higher complication rates, longer operative times, larger operative blood losses^32^ and more frequent persistent air leaks.^33^ We found no differences with regard to intra‐ or postoperative outcomes between both groups. This corroborates previous work by our group and other multicentric studies, which compared postoperative VS and VL complications rates.^34^, ^35^, ^36^, ^37^

Our study has several limitations. First, it is a retrospective study: some information may have been missing or overlooked. Second, the choice of treatment modality (VS or VL) was driven by unmeasured patient characteristics, which could induce a selection bias. Third, data about VS includes the technical learning curve. This might have initially impacted the intra‐ and postoperative outcomes. Finally, the follow‐up period was 23 months. The effectiveness of oncological treatment for NSCLC by VL or VS would be best assessed over a longer period, although a non‐negligible number of recurrences occur in the two years following surgery.^38^, ^39^ Currently, two phase III randomized clinical trials are being conducted to evaluate the surgical management of early‐stage NSCLC. One aims to analyze the noninferiority of segmentectomy for stage IA <2 cm NSCLC (JCOG0802/WJOG4607L),^40^ whereas the other compares the oncological outcomes after segmentectomy and wedge resections to those following a lobectomy for stage IA < 2 cm (NCT00499330).^41^ These long‐awaited clinical trials will shed new light on a question that has not been answered conclusively during the past decades.

In conclusion, our preliminary data shows that, in the short follow‐up period reported here, local control for patients with NSCLC <2 cm is comparable with VATS lobectomy and VATS segmentectomy.

## Disclosure

The authors report that there are no conflict of interests.

## References

[1] Ramsey HE , Cahan WG , Beattie EJ , Humphrey C . The importance of radical lobectomy in lung cancer. J Thorac Cardiovasc Surg 1969; 58 (2): 225–30. 5798227

[2] Scott WJ , Howington J , Feigenberg S , Movsas B , Pisters K . Treatment of non‐small cell lung cancer stage I and stage II: ACCP evidence‐based clinical practice guidelines (2nd edition). Chest 2007; 132 (3 Suppl): 234S–42S. 17873171 10.1378/chest.07-1378

[3] Bennett WF , Smith RA . Segmental resection for bronchogenic carcinoma: A surgical alternative for the compromised patient. Ann Thorac Surg 1979; 27 (2): 169–72. 453975 10.1016/s0003-4975(10)63261-4

[4] Miller JI , Hatcher CR . Limited resection of bronchogenic carcinoma in the patient with marked impairment of pulmonary function. Ann Thorac Surg 1987; 44 (4): 340–3. 2821937 10.1016/s0003-4975(10)63785-x

[5] Kilic A , Schuchert MJ , Pettiford BL *et al* Anatomic segmentectomy for stage I non‐small cell lung cancer in the elderly. Ann Thorac Surg 2009; 87 (6): 1662–8. 19463574 10.1016/j.athoracsur.2009.02.097

[6] Okada M , Koike T , Higashiyama M , Yamato Y , Kodama K , Tsubota N . Radical sublobar resection for small‐sized non‐small cell lung cancer: A multicenter study. J Thorac Cardiovasc Surg 2006; 132 (4): 769–75. 17000286 10.1016/j.jtcvs.2006.02.063

[7] Ginsberg RJ , Rubinstein LV . Randomized trial of lobectomy versus limited resection for T1 N0 non‐small cell lung cancer. Ann Thorac Surg 1995; 60 (3): 615–23. 7677489 10.1016/0003-4975(95)00537-u

[8] Schuchert MJ , Abbas G , Awais O *et al* Anatomic segmentectomy for the solitary pulmonary nodule and early‐stage lung cancer. Ann Thorac Surg 2012; 93 (6): 1780–7. 22483652 10.1016/j.athoracsur.2011.11.074

[9] Lutz JA , Seguin‐Givelet A , Grigoroiu M , Brian E , Girard P , Gossot D . Oncological results of full thoracoscopic major pulmonary resections for clinical stage i non‐small‐cell lung cancer. Eur J Cardio Thoracic Surg 2019; 55 (2): 263–70. 10.1093/ejcts/ezy245 30052990

[10] Asamura H , Hishida T , Suzuki K *et al* Radiographically determined noninvasive adenocarcinoma of the lung: Survival outcomes of Japan clinical oncology group 0201. J Thorac Cardiovasc Surg 2013; 146 (1): 24–30. 23398645 10.1016/j.jtcvs.2012.12.047

[11] Suzuki K , Asamura H , Kusumoto M , Kondo H , Tsuchiya R . ‘Early’ peripheral lung cancer: Prognostic significance of ground glass opacity on thin‐section computed tomographic scan. Ann Thorac Surg 2002; 74 (5): 1635–9. 12440622 10.1016/s0003-4975(02)03895-x

[12] von Elm E , Altman DG , Egger M , Pocock SJ , Gøtzsche PC , Vandenbroucke JP . The strengthening the reporting of observational studies in epidemiology (STROBE) statement: Guidelines for reporting observational studies. Int J Surg 2014; 12 (12): 1495–9. 25046131 10.1016/j.ijsu.2014.07.013

[13] Suzuki H , Morimoto J , Mizobuchi T *et al* Does segmentectomy really preserve the pulmonary function better than lobectomy for patients with early‐stage lung cancer? Surg Today 2017; 47 (4): 463–9. 27484067 10.1007/s00595-016-1387-4

[14] Charloux A , Quoix E . Lung segmentectomy: Does it offer a real functional benefit over lobectomy? Eur Respir Soc 2017; 26: 170079. 10.1183/16000617.0079-2017 PMC9488724 29070582

[15] Zheng Y‐Z , Zhai W‐Y , Zhao J *et al* Oncologic outcomes of lobectomy vs. segmentectomy in non‐small cell lung cancer with clinical T1N0M0 stage: A literature review and meta‐analysis. J Thorac Dis 2020; 12 (6): 3178–87. 32642239 10.21037/jtd-19-3802 PMC7330803

[16] Winckelmans T , Decaluwé H , De Leyn P , Van Raemdonck D . Segmentectomy or lobectomy for early‐stage non‐small‐cell lung cancer: A systematic review and meta‐analysis. Eur J Cardiothorac Surg 2020; 57 (6): 1051–60. 31898738 10.1093/ejcts/ezz339

[17] Bedetti B , Bertolaccini L , Rocco R , Schmidt J , Solli P , Scarci M . Segmentectomy versus lobectomy for stage I non‐small cell lung cancer: A systematic review and meta‐analysis. J Thorac Dis 2017; 9 (6): 1615–23. 28740676 10.21037/jtd.2017.05.79 PMC5506148

[18] Bao F , Ye P , Yang Y *et al* Segmentectomy or lobectomy for early stage lung cancer: A meta‐analysis. Eur J Cardiothorac Surg 2014; 46: 1–7. 24321996 10.1093/ejcts/ezt554

[19] Qu X , Wang K , Zhang T *et al* Long‐term outcomes of stage I NSCLC (≤3 cm) patients following segmentectomy are equivalent to lobectomy under analogous extent of lymph node removal: A PSM based analysis. J Thorac Dis 2017; 9 (11): 4561–73. 29268526 10.21037/jtd.2017.10.129 PMC5721037

[20] Howington JA , Blum MG , Chang AC , Balekian AA , Murthy SC . Treatment of stage I and II non‐small cell lung cancer: Diagnosis and management of lung cancer, 3rd ed: American college of chest physicians evidence‐based clinical practice guidelines. Chest 2013; 143 (5 Suppl): e278S–313S. 23649443 10.1378/chest.12-2359

[21] Postmus PE , Kerr KM , Oudkerk M *et al* Early and locally advanced non‐small‐cell lung cancer (NSCLC): ESMO Clinical Practice Guidelines for diagnosis, treatment and follow‐up. Ann Oncol 2017; 28: iv1–21. 28881918 10.1093/annonc/mdx222

[22] D'Amico TA . VATS Lobectomy facilitates the delivery of adjuvant docetaxelcarboplatin chemotherapy in patients with non‐small cell lung cancer. J Thorac Dis 2016; 8: 296–7. 27076920 10.21037/jtd.2016.02.36 PMC4805810

[23] Khullar OV , Liu Y , Gillespie T *et al* Survival after sublobar resection versus lobectomy for clinical stage IA lung cancer: An analysis from the National Cancer Data Base. J Thorac Oncol 2015; 10 (11): 1625–33. 26352534 10.1097/JTO.0000000000000664 PMC5798611

[24] Medbery RL , Gillespie TW , Liu Y *et al* Nodal upstaging is more common with thoracotomy than with VATS during lobectomy for early‐stage lung cancer: An analysis from the national cancer data base. J Thorac Oncol 2016; 11 (2): 222–33. 26792589 10.1016/j.jtho.2015.10.007 PMC4729646

[25] Boffa DJ , Kosinski AS , Paul S , Mitchell JD , Onaitis M . Lymph node evaluation by open or video‐assisted approaches in 11,500 anatomic lung cancer resections. Ann Thorac Surg 2012; 94: 347–53. 22742843 10.1016/j.athoracsur.2012.04.059

[26] Kneuertz PJ , Cheufou DH , D'Souza DM *et al* Propensity‐score adjusted comparison of pathologic nodal upstaging by robotic, video‐assisted thoracoscopic, and open lobectomy for non–small cell lung cancer. J Thorac Cardiovasc Surg 2019; 158: 1457–66.e2. 31623811 10.1016/j.jtcvs.2019.06.113

[27] Whitson BA , Groth SS , Andrade RS , Maddaus MA , Habermann EB , D'Cunha J . Survival after lobectomy versus segmentectomy for stage I non‐small cell lung cancer: A population‐based analysis. Ann Thorac Surg 2011; 92 (6): 1943–50. 21962268 10.1016/j.athoracsur.2011.05.091

[28] Wolf AS , Richards WG , Jaklitsch MT *et al* Lobectomy versus sublobar resection for small (2 cm or less) nonsmall cell lung cancers. Ann Thorac Surg 2011; 92 (5): 1819–25. 22051277 10.1016/j.athoracsur.2011.06.099

[29] Cao C , Chandrakumar D , Gupta S , Yan TD , Tian DH . Could less be more?‐a systematic review and meta‐analysis of sublobar resections versus lobectomy for non‐small cell lung cancer according to patient selection. Lung Cancer 2015; 89 (2): 121–32. 26033208 10.1016/j.lungcan.2015.05.010

[30] Sawabata N , Ohta M , Matsumura A *et al* Optimal distance of malignant negative margin in excision of nonsmall cell lung cancer: A multicenter prospective study. Ann Thorac Surg 2004; 77 (2): 415–20. 14759408 10.1016/S0003-4975(03)01511-X

[31] Schuchert MJ , Pettiford BL , Keeley S *et al* Anatomic segmentectomy in the treatment of stage I non‐small cell lung cancer. Ann Thorac Surg 2007; 84 (3): 926–33. 17720401 10.1016/j.athoracsur.2007.05.007

[32] Ohtsuka T , Kamiyama I , Asakura K , Kohno M . Thirty‐day outcomes after lobectomy or segmentectomy for lung cancer surgery. Asian Cardiovasc Thorac Ann 2015; 23 (7): 828–31. 26071452 10.1177/0218492315589476

[33] Suzuki K , Saji H , Aokage K *et al* Comparison of pulmonary segmentectomy and lobectomy: Safety results of a randomized trial. J Thorac Cardiovasc Surg 2019; 158 (3): 895–907. 31078312 10.1016/j.jtcvs.2019.03.090

[34] Bédat B , Abdelnour‐Berchtold E , Krueger T *et al* Clinical outcome and risk factors for complications after pulmonary segmentectomy by video‐assisted thoracoscopic surgery: Results of an initial experience. J Thorac Dis 2018; 10 (8): 5023–9. 30233876 10.21037/jtd.2018.07.42 PMC6129883

[35] Bédat B , Abdelnour‐Berchtold E , Krueger T *et al* Impact of complex segmentectomies by video‐assisted thoracic surgery on peri‐operative outcomes. J Thorac Dis 2019; 11 (10): 4109–18. 31737293 10.21037/jtd.2019.10.07 PMC6837948

[36] Bédat B , Abdelnour‐Berchtold E , Perneger T *et al* Comparison of postoperative complications between segmentectomy and lobectomy by video‐assisted thoracic surgery: A multicenter study. J Cardiothorac Surg 2019; 14 (1): 189. 31699121 10.1186/s13019-019-1021-9 PMC6836384

[37] Stamatis G , Leschber G , Schwarz B *et al* Perioperative course and quality of life in a prospective randomized multicenter phase III trial, comparing standard lobectomy versus anatomical segmentectomy in patients with non‐small cell lung cancer up to 2 cm, stage IA (7th edition of TNM staging system). Lung Cancer 2019; 138: 19–26. 31606521 10.1016/j.lungcan.2019.09.021

[38] El‐Sherif A , Fernando HC , Santos R *et al* Margin and local recurrence after sublobar resection of non‐small cell lung cancer. Ann Surg Oncol 2007; 14: 2400–5. 17505859 10.1245/s10434-007-9421-9

[39] Mohiuddin K , Haneuse S , Sofer T *et al* Relationship between margin distance and local recurrence among patients undergoing wedge resection for small (≤2 cm) non‐small cell lung cancer. J Thorac Cardiovasc Surg 2014; 147 (4): 1169–77. 24507406 10.1016/j.jtcvs.2013.11.056

[40] Nakamura K , Saji H , Nakajima R *et al* A phase III randomized trial of lobectomy versus limited resection for small‐sized peripheral non‐small cell lung cancer (JCOG0802/WJOG4607L). Jpn J Clin Oncol 2009; 40 (3): 271–4. 19933688 10.1093/jjco/hyp156

[41] Altorki N , Kohman LJ , Veit JL , You YN , Boughey JC . Limited resection as a cure for early lung cancer: Time to challenge the gold standard? Bull Am Coll Surg 2015; 100: 57–8. 26031124

